# Temporomandibular joint (TMJ) disorders prevalence and awareness of appropriate clinical practices, among Al-Madinah community in Saudi Arabia

**DOI:** 10.12688/f1000research.104272.1

**Published:** 2022-04-06

**Authors:** Albraa Alolayan, Shayma S. Alsayed, Ruwaa M. Salamah, Khadija M. Ali, Mashael Alsousi, Shadia Elsayed

**Affiliations:** 1 Department of Oral and Maxillofacial Surgery, College of Dentistry, Taibah University, Almadinah Almunawwarrah, Madinah, Saudi Arabia; 2 Dentistry, Ministry of Health, Al Madinah Al Munawwarah, Madianh, Saudi Arabia; 3 Department of Oral and Maxillofacial Surgery, Faculty of Dental Medicine for Girls, Al Azhar University, Cairo, Egypt

**Keywords:** TMJ, prevalence, awareness, severity, Almadinah Almunawwarrah

## Abstract

**Background: ** Painful temporomandibular joint disorders (TMDs) are of musculoskeletal origin and are considered the most common cause of non-odontogenic pain in the orofacial region.  The purpose of this study was to investigate the prevalence and awareness of temporomandibular joint (TMJ) disorders in Almadinah Almunawwarah community.

**Methods**: An observational cross-sectional study with convenience sampling was conducted. A modified version of Fonseca's questionnaire was employed. The questionnaire asked about the participant's personal information, if they thought they had TMDs, and who to visit for therapy if necessary. These were followed by 10 items from Fonseca's questionnaire, each with a three-point scale.

**Results**: The questionnaire was completed by 598 people. Females made up 57.1% of the participants. TMDs were present in 61% of the population, with varying degrees of severity. Males (44.3%) were less affected than females (55.7%). The difference, however, was not statistically significant (P = 0.354). Out of the 61% TMDs Positive patients, 74.1% had mild TMDs symptoms, while 20.8% and 5.1%, respectively, had moderate and severe TMDs symptoms (P = 0.05). The severity of the symptoms was unaffected by demographic data (P > 0.05). Only 40% seek care, with 64.6% selecting for a dentist and 24.6% preferring for an orthopaedic specialist (P= 0.008).

**Conclusions**: Participants from Al-Madinah had a greater prevalence of mild TMDs. The majority of the participants had no idea who to go to for treatment. The findings of this study highlight the importance of educational activities to enhance public awareness. Fonseca's Anamnestic Index could also be considered as a useful instrument for early identification and measuring the severity of TMDs in the general population.

## Introduction

Temporomandibular disorders (TMDs) is a term used to describe a set of musculoskeletal and neuromuscular diseases characterized by localized discomfort in the pre-auricular and face regions, as well as deviations or restrictions in mandibular joint movement, in addition to joint noises. ^1^ TMDs are multifactorial, caused by stress, trauma and malocclusion. ^2^ Painful TMDs are of musculoskeletal origin and are considered the most common cause of non-odontogenic pain in the orofacial region. ^3^^–^^5^ The most prevalent symptom of TMD is tenderness of the masticatory muscles when palpated. ^6^^,^^7^

The prevalence of TMDs was twice higher in women compared to men, ^8^ also pain intensity was greater in women. ^9^ According to a previous report, 75% of the participants exhibited one TMD sign and 33% had one TMD symptom. ^10^ Another study concluded that 50–75% of the population had TMD signs at some moment in their lives. ^11^ One study showed that only 39% participants had sought treatment for pain related to TMDs. ^12^ Patients with TMDs symptoms tend to consult first with general medical practitioners due to the availability and financial feasibility in nearly six countries. 27% of children and adolescents in Saudi Arabia were found to have TMDs.

For our patients, temporomandibular joint (TMJ) disorders still cause difficulty in diagnosis and conflicts between different specialties. Some individuals seek treatment from different specialized clinics such as ENT (ear, nose and throat) or neurosurgery clinics, and therefore the goal of this study was to establish the prevalence, awareness, and severity of TMJ disorders in our Almadinah Almunawwarah community.

## Methods

### Study design and setting

This study was designed to be an observational cross-sectional study with convenience sampling technique. The Taibah University Dental College Ethical Committee approved the study protocol (TUCDREC/20200219/MIAlsousi), and all participants’ identities were kept anonymous. The study followed the Helsinki Declaration on Human Research Studies. The patients were selected from the dental college and the hospital at Taibah University in Al-Madinah, Saudi Arabia. Following an explanation of the study’s aims and objectives, patients who agreed to participate in the study signed an informed consent form.

### Inclusion and exclusion criteria

All patients with systemic disease associated with TMJ like rheumatoid arthritis, regular use of medication such as anti-anxiety or antidepressants, history of TMJ surgery, wearing removable denture or splints, psychological problems and/or with incomplete questionnaire form were excluded from the study.

Response bias was reduced by keeping the questions simple and short, as in the verified Fonseca questionnaire, which has structured proper length and understandable language, and by limiting the patient experience to the previous three months.

Using a sample size calculator with a confidence level of 99% and estimated the entire Madinah population to be above one million, the sample size was calculated to be 523 participants. To avoid missing or insufficient data, we extend the total to 598. Patients seeking dental treatment were recruited through the Oral and Maxillofacial Surgery clinics at Taibah University, Almadinah Almunawwarah, in Saudi Arabia.

### Instrument for data collection

In order to estimate Fonseca’s Anamnestic Index (FAI), a modified Fonseca’s questionnaire was utilized. The original Fonseca’s questionnaire was proposed by Fonseca *et al* ^13^ in Brazilian Portuguese.

The questionnaire included questions about participant’s demographic data, if the participant thought he/she is suffering from TMDs, and whom to seek for a treatment if he/she needed to. These questions were followed by 10 questions of Fonseca’s questionnaire with three points scale, and it was distributed to patients in an Arabic version. For each question, the participants were instructed that just one answer should be marked: “yes” (10 points), “no” (0 points), and “maybe” (5 points). Based on the sum of their points, the individuals were classified as TMD free (0–15), mild TMD (20–40), moderate TMD (45–60), and severe TMD (70–100). The FAI has been usually applied in Brazilian studies to measure the severity of TMDs. ^9^^,^^14^ A copy of the questionnaire can be found in the Extended data.

### Statistical analysis

The data was evaluated using a social science statistical software (SPSS version 25). Means, standard deviation, and percentages were used to show descriptive data. It was followed by Chi-square test for nominal outcomes and Student’s t-test for continuous study variables. Significance level was set at *P* value of 0.05.

## Results

**Figure 1.**
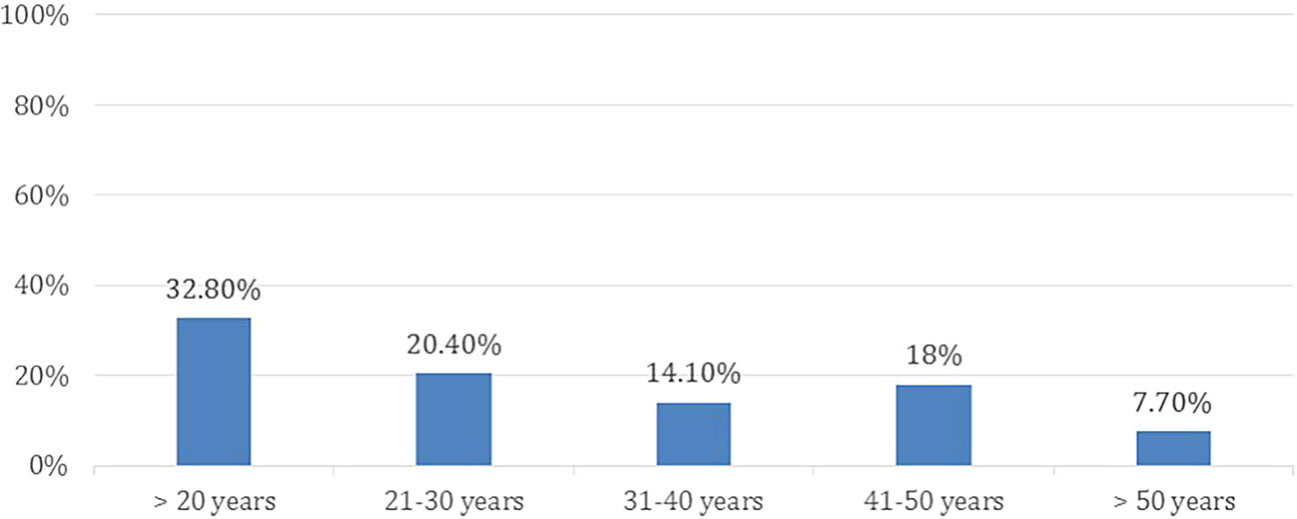
Age group distribution.

**Figure 2.**
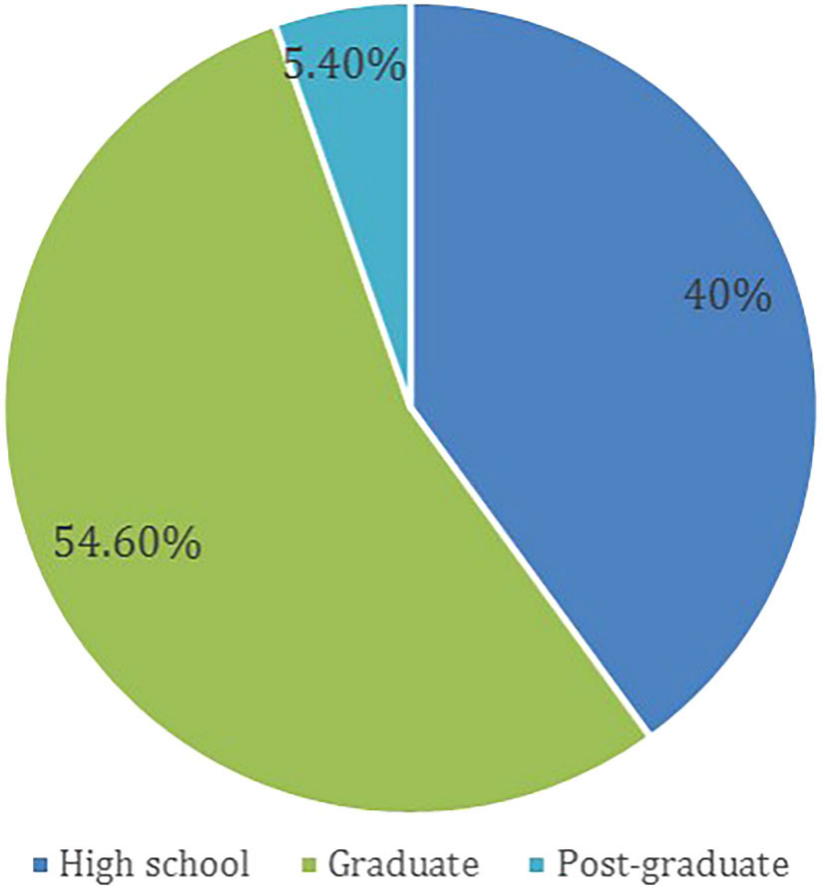
Level of education.

A total of 598 participants answered the questionnaire, based on the above-mentioned exclusion criteria, 80 (13.4 %) participants were removed from the study. Thus, leveeing a total of 518 (86.6%) recruited participants, females made up 57.1% of the participants. The age group distribution of participants and the level of education are presented in Figure 1 and Figure 2, respectively.

**Figure 3.**
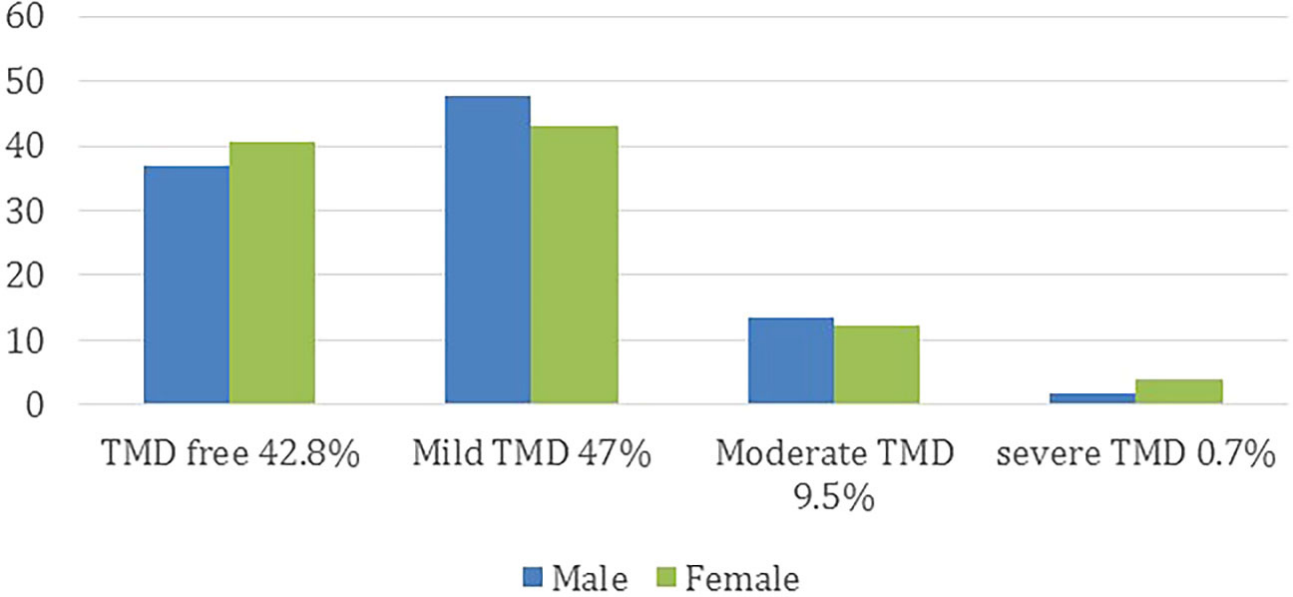
The level of TMDs in regard to gender distribution.

Based on the participants’ answers on Fonseca’s questionnaire, 61% had TMDs with variable degree of severity. Male group (44.3%) was less than female group (55.7%). The difference, however, was not statistically significant ( *P* = 0.354). On the other hand, there was a significant difference in the positive responses to the questions of whether or not the participants had TMDs (12.5 %), and the participants whom actually suffering from TMDs according to Fonseca’s questionnaire ( *P* < 0.05). The level of TMDs in regard to gender distribution is presented in Figure 3.

Out of the 61% TMDs positive patients, 74.1% had symptoms of mild TMDs, while the moderate and severe symptoms were 20.8% and 5.1%, respectively ( *P* < 0.05). However, the demographic data showed no effect on the severity of the symptoms ( *P* > 0.05).

In regards whom to seek for treatment, 51.6% of the sample chose a dentist while 41.8% chose an orthopedic specialist ( *P* > 0.05). Based on participants who suffered or suffering from TMDs, only 40% sought out treatment, 64.6% of them chose a dentist while 24.6% chose orthopaedic specialist ( *P* ≤ 0.008).

The symptoms mostly associated with TMDs ranged from behavioral changes and quick fatigue of the mastication muscles, to frequent headache or pain related to the TMJ or the neck, reaching to TMJ stiffness and clicking or even limitation in mouth opening. Table 1 summarizes the frequencies of the most common symptoms.

**Table 1.** The frequency of the most common symptoms for patient with TMDs.

Symptom	Frequency (n = 518)	Percentage
Limited mouth opening	12	2.3%
Limited side-to-side movement	7	1.4%
Muscles of mastication fatigue	29	5.6%
Frequent headaches	211	40.7%
Neck pain or stiffness	55	10.6%
Pain in the ear or TMJ area	44	8.5%
TMJ clicking	102	19.7%
Temper behavior	151	29.2%

## Discussion

The study evaluates the prevalence and awareness of TMD amongst the population of Al-Madinah to produce sufficient data on TMD, helps to find crucial part of planning programs to educate and raise awareness through the population. The study showed that TMDs were present in 61% of the population. Many studies employed the Fonseca questionnaire to diagnose and grade the severity of TMDs. TMD is divided into four categories: minor, moderate, severe, and non-TMD. ^15^ It has a number of advantages, including quick application, low cost, minimal inconsistency, and self-administration. ^16^ The Fonseca questionnaire was used in this study to assess the prevalence of TMD symptoms and manifestations in the Almadianh region of Saudi Arabia. It is used to gather data from a wider number of people in a short period. The most significant advantage of employing this questionnaire is that it has no bearing on the investigator and is straightforward. ^17^

More than half of the participants had some level of TMDs, which has been associated with headache. In regard, frequent headaches were associated with 40% of the participant, patients with TMD are five times more likely to report headaches. ^8^ The results are related to a study done in Brazil in 2018 which over two third of the adolescents had headache/migraine and 36 percent of them connected it with TMD. ^18^

Though TMD is suggested to have a higher frequency and severity in females than in males, and females were reported to have a greater sensitivity to pain ^19^ which has been connected to hormonal, psychological, and even neurological changes, ^8^^,^^9^^,^^20^ no conclusive results achieved to the date. In this study TMD male patients (44.3%) were less than female patients (55.7%). However, the difference is not significant, also in a similar study done in Al-Badar Dental College mentioned that men (49.14%) had higher free of TMD symptoms than women (44.78%) but this difference was also not statistically significant. ^20^

Although more than 70% of the TMD positive participants have mild TMD, only 51.6% of the sample knows who to see for TMD therapy, and 40% of TMD patients seek treatment. TMD treatment varies from one physician to another. It ranges from patient education and the development of new habits such as self-massage, hot and cold packs, diet and nutrition instruction, and muscle exercises, which have been shown to have a high level of success and improvement, to the need for invasive and high-technical or invasive surgical treatments. ^1^^,^^21^^,^^22^ According to Reynaldo, 91.7% of patients responded well to conservative treatment. There was no TMD recurrence in the majority of patients who could be examined four to six years after treatment completion. ^23^ The current study’s findings highlight the importance of public education and emphasize the limitations of generalizing the findings to other sectors of the health clusters in our community’s population. ^24^

## Conclusion

Based on this study, participants from Al-Madinah showed higher prevalence in mild degree of TMDs. Most of the participants were lacking the knowledge of TMDs and whom to seek for a treatment. The results from this study emphasize the need for educating programs to raise awareness through the population, and to consider Fonseca’s anamnestic index as a valuable tool for early identification and severity assessment of TMDs in the general population.

## Data availability

### Underlying data

Figshare: Data for TMJ disorders prevalence and awareness of appropriate clinical practices, among Al-Madinah community in Saudi Arabia.sav https://doi.org/10.6084/m9.figshare.17215940 ^25^

This project contains the following underlying data

- Data for TMJ disorders prevalence and awareness of appropriate clinical practices, among Al-Madinah community in Saudi Arabia.sav (raw data in Arabic)

Data are available under the terms of the Creative Commons Attribution 4.0 International license (CC-BY 4.0).

Figshare: Data for TMJ disorders prevalence and awareness of appropriate clinical practices, among Al-Madinah community in Saudi Arabia.sav. https://doi.org/10.6084/m9.figshare.19086635.v2 ^26^

This project contains the following underlying data

- Data for TMJ disorders prevalence and awareness of appropriate clinical practices, among Al-Madinah community in Saudi Arabia.sav (raw data in English)

Data are available under the terms of the Creative Commons Attribution 4.0 International license (CC-BY 4.0).

### Extended data

Figshare: Questionnaire. https://doi.org/10.6084/m9.figshare.19292474 ^27^

This project contains the following extended data

A copy of the questionnaire (in Arabic and the English version).

Data are available under the terms of the Creative Commons Attribution 4.0 International license (CC-BY 4.0).
