# Supplementary material for: Temporomandibular joint (TMJ) disorders prevalence and awareness of appropriate clinical practices, among Al-Madinah community in Saudi Arabia
Source: F1000Res. 2022 Apr 11;11:395. Originally published 2022 Apr 6. [Version 2] doi: 10.12688/f1000research.104272.2 (PMC10157292; doi:10.12688/f1000research.104272.2)
Supplement: Peer review history

# Peer review: Reviewer response for version 2

**DOI:** 10.5256/f1000research.126287.r134580

**Published:** 2022-04-13

**Authors:** Essam Ahmed Al‐Moraissi

**Affiliations:** 1 Department of Oral and Maxillofacial Surgery, Faculty of Dentistry, Thamar University, Dhamar, Yemen

*Update 07/07/2022 – We have added a conflict of interest statement that was not disclosed before this report was published. This statement can be found below this report under the heading ‘Competing Interests’.*

 Thank you for your revised version of your manuscript. However, before considering the paper for indexing, I have the following concerns: 

1. Authors stated in the inclusion criteria that "All adult males and females who visited Taibah University’s Oral and Maxillofacial Surgery clinics in Almadinah Almunawwarah, Saudi Arabia, in the previous two years were included in the study." 
  My question is what are the purposes of visiting participants? What did they seek treatments for? Were they asked about treatments for TMDs? 
2. Since this is an observational cross-sectional study,  authors should clarify if this study was performed retrospectively or prospectively and should state this information in the methods and abstract. 
3. It's clear that sample size calculation was not reliable and convincing because sample size calculation for cross-sectional study requires statistical formula, the prevalence of an indicator (which should be estimated from the previous published similar studies), level of significance, correlation coefficient, etc.
  Based on methods authors stated clearly that "All adult males and females who visited Taibah University’s Oral and Maxillofacial Surgery clinics in Almadinah Almunawwarah, Saudi Arabia, in the previous two years were included in the study."
  So, all patients that visited the clinic within 2 years have been included, is this means no actual sample size calculation was performed? Please clarify. Otherwise, the authors should address these points as a study limitation. 
4. Based on the methodology, the authors included all patients who visited the Department at a specific time. Also, based on the Arabic Questionnaire,  and the author's exclusion criteria,  they excluded some patients. So, it's mandatory to clarify the total number of participants who were initially recruited (based on sample size calculation,  in the case this has been done prospectively) and agreed to participate, then the number of patients who were excluded with reasons using flow diagram. 
5. In Conclusion, the authors should address the primary aim of the study which estimated the prevalence of TMDs in the City. As this study is a cross-section study,  the causation between the risk factor and outcome is impossible to assess. Then I think, some part of the conclusion is not supported by the findings (Fonseca's Anamnestic Index could also be considered a useful instrument for early identification and measuring the severity of TMDs in the general population.) 
6. The authors stated in the exclusion criteria those patients who wear removable dentures or splints were excluded, please give an explanation 
7. Another problem could be introducing a bias within the study, and difficulties for patients to fill and complete the questionnaire.
8. Title: abbreviation of (TMJ) should be removed from the title. The title should be short more focused and mention the study design 

Is the work clearly and accurately presented and does it cite the current literature?

Yes

If applicable, is the statistical analysis and its interpretation appropriate?

Partly

Are all the source data underlying the results available to ensure full reproducibility?

No

Is the study design appropriate and is the work technically sound?

No

Are the conclusions drawn adequately supported by the results?

Partly

Are sufficient details of methods and analysis provided to allow replication by others?

No

Reviewer Expertise:

Oral and Maxillofacial Surgery

I confirm that I have read this submission and believe that I have an appropriate level of expertise to confirm that it is of an acceptable scientific standard, however I have significant reservations, as outlined above.

# Response

**Published:** 2022-06-30

**Authors:** Shadia Elsayed

**Affiliations:** Al Azhar University, Egypt

Thank you for your feedback, which provided us with another opportunity to edit the article.

1. Authors stated in the inclusion criteria that "All adult males and females who visited Taibah University’s Oral and Maxillofacial Surgery clinics in Almadinah Almunawwarah, Saudi Arabia, in the previous two years were included in the study." My question is what are the purposes of visiting participants? What did they seek treatments for? Were they asked about treatments for TMDs? 
**Answer:** Thank you for your input. The study included individuals who came to the TUDC for routine dental screening, with the goal of determining the prevalence of TMJ problems in this specific demographic.
2. Since this is an observational cross-sectional study, authors should clarify if this study was performed retrospectively or prospectively and should state this information in the methods and abstract. 
**Answer:** Thank you for your input. The study was conducted as a prospective observational study, and this is now addressed in the methodology and abstract.
3. It's clear that sample size calculation was not reliable and convincing because sample size calculation for cross-sectional study requires statistical formula, the prevalence of an indicator (which should be estimated from the previous published similar studies), level of significance, correlation coefficient, etc.
**Answer:** I appreciate your input. The study sample calculation was determined using the OpenEpi tool kit application with a confidence level of 99 percent and estimated the entire Madinah population to be above one million and estimated prevalence indicator of 27% (Al-Khotani, A., Naimi-Akbar, A., Albadawi, E. *et al.* Prevalence of diagnosed temporomandibular disorders among Saudi Arabian children and adolescents. J Headache Pain 17, 41 (2016). https://doi.org/10.1186/s10194-016-0642-9) and the sample size was calculated to be 523 participants. To avoid missing or insufficient data, we extend the total to 598. 
  Based on methods authors stated clearly that "All adult males and females who visited Taibah University’s Oral and Maxillofacial Surgery clinics in Almadinah Almunawwarah, Saudi Arabia, in the previous two years were included in the study." So, all patients that visited the clinic within 2 years have been included, is this means no actual sample size calculation was performed? Please clarify. Otherwise, the authors should address these points as a study limitation. 
**Answer:** Thank you for your comment. The study was conducted during the last two years, and the sample was drawn from clinic visitors on a daily basis.
4. Based on the methodology, the authors included all patients who visited the Department at a specific time. Also, based on the Arabic Questionnaire, and the author's exclusion criteria, they excluded some patients. So, it's mandatory to clarify the total number of participants who were initially recruited (based on sample size calculation, in the case this has been done prospectively) and agreed to participate, then the number of patients who were excluded with reasons using flow diagram. 
**Answer:** Thank you for your comment. The study included A total of 598 participants answered the questionnaire, based on the mentioned exclusion criteria, 80 (13.4 %) participants were removed from the study. Thus, leveeing a total of 518 (86.6%) recruited participants
5. In Conclusion, the authors should address the primary aim of the study which estimated the prevalence of TMDs in the City. As this study is a cross-section study, the causation between the risk factor and outcome is impossible to assess. Then I think, some part of the conclusion is not supported by the findings (Fonseca's Anamnestic Index could also be considered a useful instrument for early identification and measuring the severity of TMDs in the general population.) 
**Answer:** Thank you for your comment. Conclusion section has been updated in accordance with your thoughtful advice.
6. The authors stated in the exclusion criteria those patients who wear removable dentures or splints were excluded, please give an explanation.
**Answer:** In order to assess the actual prevalence among the population that did not get prosthodontic devices like splints, we eliminated patients receiving splint therapy and removable prosthodontics, which could be a contributing confounding cause of TMJ adaptive modifications.
7. Another problem could be introducing a bias within the study, and difficulties for patients to fill and complete the questionnaire.
**Answer:** thank you for your comment. The questionnaires were filled out by well-trained two investigators who collect data from patients and contribute to the current study's authorship. 
8. Title: abbreviation of (TMJ) should be removed from the title. The title should be short more focused and mention the study design 
**Answer:** Thank you for your comment. The title has been updated in response to your helpful suggestion.

# Peer review: Reviewer response for version 1

**DOI:** 10.5256/f1000research.114358.r129949

**Published:** 2022-04-06

**Authors:** Essam Ahmed Al‐Moraissi

**Affiliations:** 1 Department of Oral and Maxillofacial Surgery, Faculty of Dentistry, Thamar University, Dhamar, Yemen

*Update 07/07/2022 - We have added a conflict of interest statement that was not disclosed before this report was published. This statement can be found below this report under the heading ‘Competing Interests’.*

 Thank you for giving me this chance to review this paper. My comments are: 

1. Authors completely failed to state the rationale of the study (why it's important to do this study) or show the size of the gap in the literature. Unfortunately, the indexing of this study will not add any new material to the existing literature. 
2. Authors did not state the inclusion criteria completely, only mentioned the exclusion criteria. So authors should state inclusion criteria based on PICOS criteria. 
3. Authors did not present adequately information about how they calculated the sample size.
4. Authors should explain why they chose a modified version of Fonseca's questionnaire.
5. The generalizability of this study is not applicable. 
6. Authors should include others predictors variables to be analysed type of TMDs if myogenous or arthrogenous, chronicity, signs and symptoms of TMDs such as clicking, muscles tenderness, headache, limited mouth opening etc.
7. Authors did not clarify who performed the assessment.
8. This study is based on a survey so its mandatory to clarify the limitations of this study. 

Is the work clearly and accurately presented and does it cite the current literature?

Yes

If applicable, is the statistical analysis and its interpretation appropriate?

Partly

Are all the source data underlying the results available to ensure full reproducibility?

No

Is the study design appropriate and is the work technically sound?

No

Are the conclusions drawn adequately supported by the results?

Partly

Are sufficient details of methods and analysis provided to allow replication by others?

No

Reviewer Expertise:

Oral and Maxillofacial Surgery

I confirm that I have read this submission and believe that I have an appropriate level of expertise to state that I do not consider it to be of an acceptable scientific standard, for reasons outlined above.
